# Early molecular diagnosis of BRAF status drives the neurosurgical management in BRAF V600E-mutant pediatric low-grade gliomas: a case report

**DOI:** 10.1186/s12887-022-03711-6

**Published:** 2022-11-29

**Authors:** Gianluca Piccolo, Antonio Verrico, Giovanni Morana, Gianluca Piatelli, Patrizia De Marco, Valentina Iurilli, Manila Antonelli, Gabriele Gaggero, Antonia Ramaglia, Marco Crocco, Samuele Caruggi, Claudia Milanaccio, Maria Luisa Garrè, Marco Pavanello

**Affiliations:** 1 Department of Neurosciences, Rehabilitation, Ophthalmology, Genetics, Maternal and Child Health, Università Degli Studi Di Genova, Genoa, Italy; 2 Neuro-Oncology Unit, IRCCS Istituto Giannina Gaslini, Genoa, Italy; 3 Department of Neuroscience “Rita Levi Montalcini”, University of Turin, Via Cherasco 15, 10126 Turin, Italy; 4 Neurosurgery Department, IRCCS Istituto Giannina Gaslini, Via G. Gaslini 5, 16147 Genoa, Italy; 5 UOC Medical Genetics, IRCCS Istituto Giannina Gaslini, Genoa, Italy; 6 Pharmacy Unit, IRCCS Istituto Giannina Gaslini, Genoa, Italy; 7 Department of Radiological, Oncological and Anatomo-Pathological Sciences, University Sapienza, Viale Regina Elena 324, 00161 Rome, Italy; 8 Pathology Unit, IRCCS Istituto Giannina Gaslini, Genoa, Italy; 9 Neuroradiology Unit, IRCCS Istituto Giannina Gaslini, Genoa, Italy

**Keywords:** Case report, Vemurafenib, Neurosurgery, Brain tumors, BRAF

## Abstract

**Background:**

To date, this is the only report showing with close and consecutive magnetic resonance images the extremely rapid response of two types of pediatric low-grade gliomas (PLGG) to vemurafenib and its impact on the surgical approach.

**Cases presentation:**

We report two cases of symptomatic PLGG treated with vemurafenib, a BRAF inhibitor: in a 12-year-old girl it was used as first-line medical treatment, reducing the tumor by 45% within a month and stabilizing to 76% after a year; in a 3-year-old boy with no improvement after SIOP LGG 2004 Protocol, vemurafenib induced in only one week a 34% shrinkage and solved the hydrocephalus, avoiding surgical operation.

**Discussion and conclusions:**

Our cases demonstrate how an early molecular diagnosis of BRAF mutations through the neurosurgical biopsy is essential to promptly start targeted therapies., whose effect can influence both therapeutic and surgical decisions, hopefully reducing the occurrence of second neurosurgery with associated risks of neurological sequelae.

## Background

Pediatric low-grade gliomas (PLGG) are the most frequent brain tumors in children [1]. The most common genetic aberrations in PLGG result in constitutive activation of a proto-oncogene of the mitogen-activated protein kinase pathway BRAF (B-Raf, a serine/threonine-protein kinase) [2–5], encoding for a protein involved in cell signaling and growth. In particular, the missense *BRAF V600E* is an oncogenic driver mutation that results in a loss of inhibition and gives BRAF oncogenic potential. PLGGs with this variant show a good response to targeted approaches such asvemurafenib (PLX4032), an orally available selective BRAF inhibitor [6]. Vemurafenib as single agent has proved to determine both radiological and clinical response in pediatric gangliogliomas [7–9], optic pathway gliomas [10], desmoplastic infantile astrocytoma [5], and high-grade gliomas [11, 12].

Here we describe the extremely rapid radiological response to vemurafenib shown by two *BRAF-*positive mutant PLGGs and the consequent effect on surgical decisions.

## Cases presentation

**Fig. 1:**
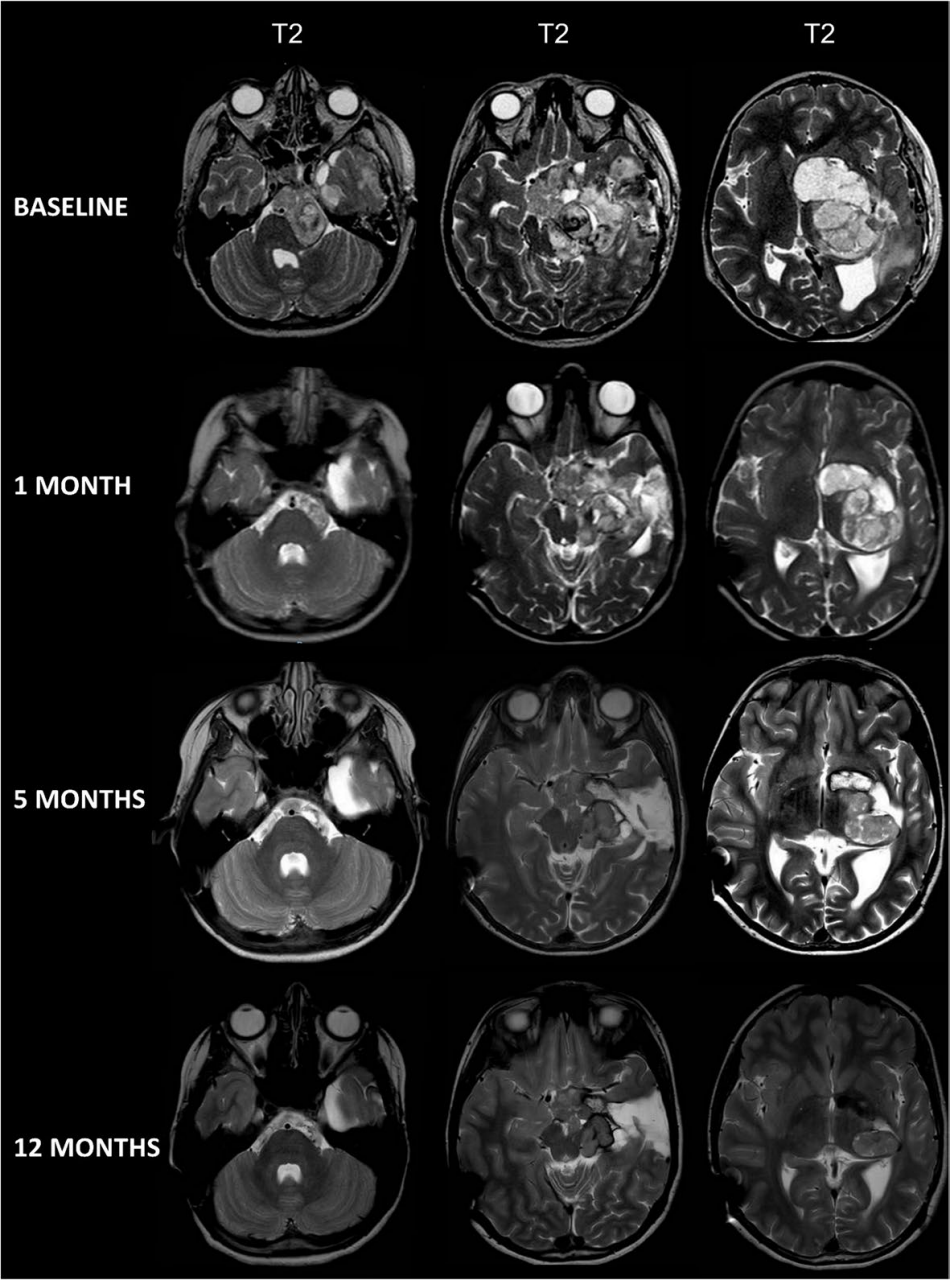
Brain MRI of the 12-year-old girl with ganglioglioma. T2-weighted axial sequences at baseline (after biopsy) show a heterogeneous mass with epicenter in the hypothalamic-chiasmatic region, prominent involvement of the left basal ganglia and thalamus, and caudal exophytic extension with midbrain compression. Follow-up MRI at 1 month from starting vemurafenib shows quick and evident shrinkage (-45%, according to RANO criteria) with prominent regression of the left prepontine component. Mass reduction continues at 5 months (-70%) and is stable at 12 months (-76%)

A 12-year-old girl presented with progressive motor impairments, right-sided hemiparesis, and homonymous right hemianopsia. Brain MRI at admission demonstrated an extensive intra-axial lesion involving the left basal ganglia, hypothalamus, and diencephalic-mesencephalic junction, with exophytic components extending into the pre-pontine cistern and to the left temporal-mesial region. The mass effect caused a non-compensated hydrocephalus, urgently treated with a ventriculoperitoneal shunt. A subtotal resection was then performed, leading to the histological diagnosis of Ganglioglioma. The option of a second surgery with the objective of a extended debulking was also considered, but when Sanger sequencing of tumor tissue identified the *BRAF V600E* variant, a targeted medical approach was preferred. After specific consent was obtained from our Institution and the family, vemurafenib was started adopting a dose escalating protocol, with initial dose of 480 mg/m^2^ daily, progressively increased up to 1080 mg/m^2^ strictly monitoring cutaneous and cardiological adverse effects. In two weeks motor skills clearly improved. A brain MRI performed one month after treatment showed a 45% shrinkage of the mass according to RANO criteria. It raised up to 70% in 5 months and stabilized to 76% after one year (Fig. 1). Clinically, the patient achieved better balance control, an almost complete resolution of the right arm paresis, and gait improvement. Several adverse effects occurred during the therapy, mainly involving the skin (photosensitivity with diffuse maculo-papular rash – grade 3, according to the Common Terminology Criteria for Adverse Events 4.0 – and grade 2 alopecia) but also grade 1 hypercholesterolemia, neutropenia, and QT prolongation were reported. In order to reduce these effects, after 21 months the therapy was changed to the combination of dabrafenib and trametinib, currently ongoing.

**Fig. 2:**
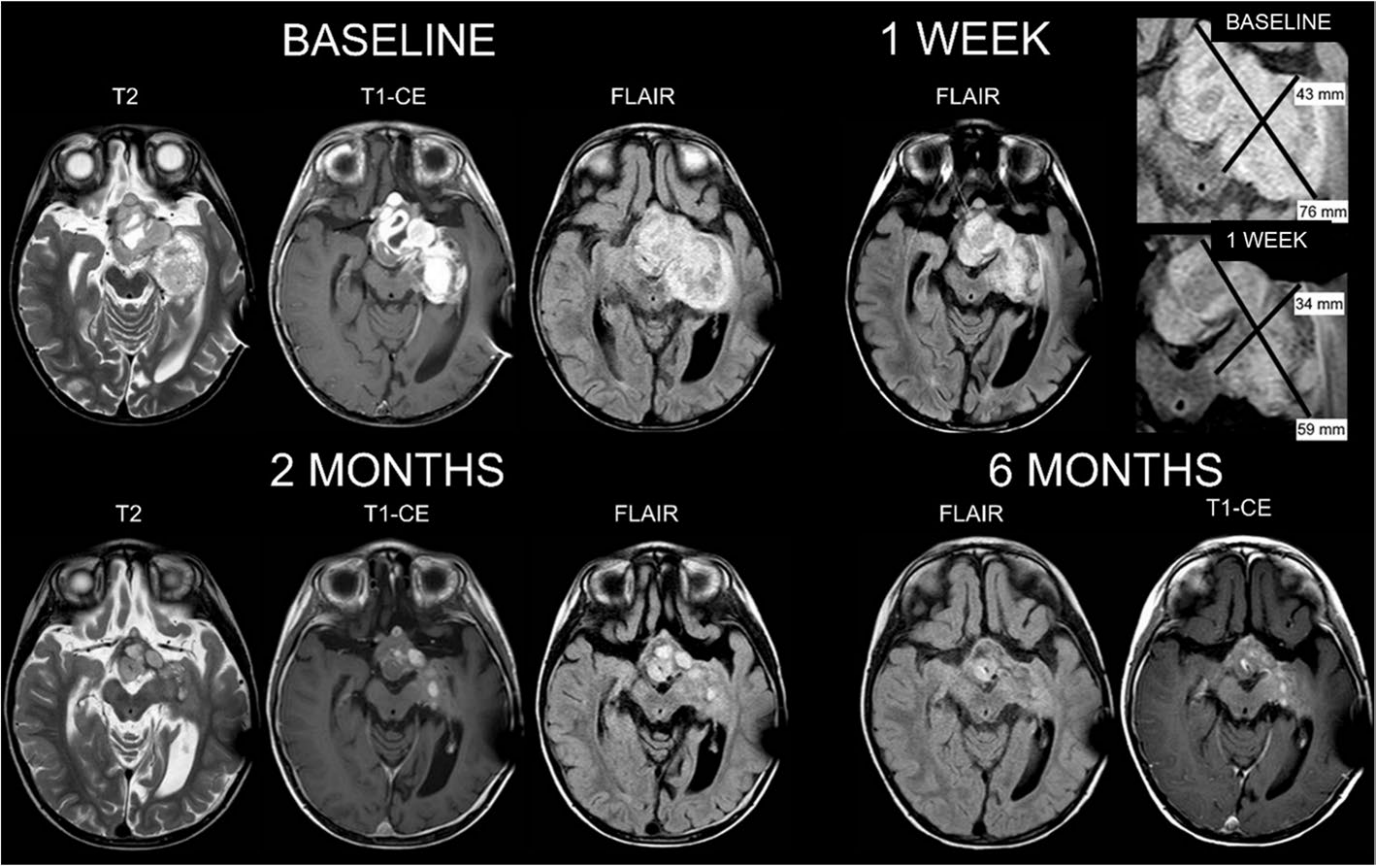
Brain MRI of the 3-year-old boy with pilocytic astrocytoma. Baseline MR and after 2 months in T2-weighted, contrast-enhanced T1-weighted, and FLAIR (FLuid Attenuated Inversion Recovery) show a 61% shrinkage of the mass. MRI at 1 week of therapy (FLAIR) shows a 38% reduction (see small frames, main diameters are reported in millimeters)

The second case is the one of a 3-year-old boy who presented with blurred vision, bilateral nystagmus, hemiplegic gait, and motor dysfunction of the right extremities. Brain MRI showed an optic pathway/hypothalamic lesion extending along the left optic tract to the basal ganglia. A biopsy of the lesion and ventriculoperitoneal shunting were performed. A Pilocytic Astrocytoma was diagnosed and a first-line treatment according to SIOP LGG 2004 Protocol started. At the end of the induction phase, the tumor had increased and ascites, hydrocephalus, and worse visual impairment occurred. Molecular testing showing *BRAF V600E* mutation on the initial tumor biopsy was obtained; therefore, the surgical option was postponed and therapy with vemurafenib started (750 mg/m^2^ daily, progressively increased to 1100 mg/m^2^). After only 3 days, eyesight improved and a better muscle tone was found. An MRI performed 1 week later showed a 34% volume reduction; ascites decreased too. Brain MRI at two and six months of treatment proved a further shrinkage of 57% and 65% to baseline (Fig. 2). The child quickly and fully recovered, regaining upper limb motor functions, good coordination, improving vision in his left eye and walking abilities. No adverse effects were reported, except for grade 1 hypercholesterolemia and the patient has continued vemurafenib for over 36 months to date. Both patients did not experience any endocrinological dysfunction after starting treatment.

## Discussion and conclusions

First-line treatment for PLGG consists of surgery in 80% of all cases. Non-surgical treatment (chemotherapy, radiotherapy) is preferred when the lesion is unresectable, gross total resection cannot be achieved, in case of secondary lesions at radiological follow-up, hemorrhagic cysts, or if threatening neurological symptoms occur [13]. The choice of primary non-surgical treatment is mainly subordinate to the age of the patient, tumor size, and location [14]. Standard first-line chemotherapy for PLGG achieves a tumor reduction in 40–50% of patients and a 5-year Progression-Free Survival (PFS) of 46% [15]. Radiotherapy produces a higher PFS with the cost of worse side effects: this justifies the preference for chemotherapy in younger children [16]. Chemotherapy administration aims at delaying or obviating radiotherapy, thus minimizing its cognitive, endocrine, and vascular consequences [14]. Notably, *BRAF V600E* PLGG is less responsive to standard first-line chemotherapy and radiotherapy [15, 16].

We describe the rapidity of both clinical and radiological improvement in two patients affected by *BRAF V600E* PLGG once treated with vemurafenib, highlighting how it favorably influenced the subsequent therapeutic and surgical decisions. We aimed to avoid further surgery, in order to reduce the risk of secondary neurological sequelae, as well as limit radiotherapy because of its well-known late effects. Importantly, in both cases a radical surgical approach would have been hard to perform due to the anatomical localization of the masses and the high risk of permanent impairment.

In the girl, vemurafenib was used as medical frontline therapy, obtaining a sudden reduction in mass dimension and neurological amelioration in only one month. The younger boy was first treated with conventional chemotherapy for PLGG, but after an initial response, disease progression occurred causing a misfunction of the ventriculoperitoneal shunt, hydrocephalus, and ascites. Also in this case, vemurafenib induced a very rapid clinical, neurological and visual improvement, corresponding to radiological reduction detected by MRI after only six days of treatment, thus avoiding a surgical revision of the shunt. The compliance to therapy was very strict in both patients. Notably, we started with lower doses and adopted an escalating approach, with the aim of testing its tolerability and the reducing occurrence of side effects. Importantly, both the patients presented quick shrinkage already in the first days of therapy, before reaching the full dose of medication, thus raising interest as future field of research in finding its lower effective dose.

Typically, in PLGG the incidence of *BRAF V600E* is age-dependent, with a predilection for infants and young children (< 3 years of age), tumors have a higher tendency for multicentricity and in some case reports a certain propensity toward a more aggressive behaviour is described [17–20]. Ho et al. demonstrated that *BRAF V600E* -mutant diencephalic PLGGs have a more aggressive clinical course, especially in children under the age of 13 years (5 years PFS 9% *versus* 46% in *BRAF* wildtype) [21].

Several authors have shown the benefits of BRAF inhibition in controlling and reducing LGG progression after first-line treatment [1, 3, 12–14, 21, 22], but only in a few patients it has been used as the first choice at diagnosis instead of standard therapies. In particular, Del Bufalo et al. assessed approximately 60% response to vemurafenib as first-line therapy in a small cohort of 6 patients, with low to mild toxicity [22].

A Phase I Study including 19 patients reported fewer adverse effects in children than in adult patients, for equivalent doses. Of note, only one progressed on therapy, while 4 of the 14 that came off drug progressed in the following year (three within four months) [23]. Unfortunately, vemurafenib did not show the same efficacy in high-grade gliomas, with considerable variability related to the histologic subtype [24].

Van Tilburg et al. reported on how BRAF inhibitors can induce tumor mass shrinkage even when used twice in the same subject: the first administration of vemurafenib for desmoplastic infantile astrocytoma needed three months to determine clinical improvement and volume reduction; treatment was discontinued for a year, then restarted after evidence of disease progression. The second cycle took only three weeks to produce a clinical and radiological response [5].

In a recent study on 56 PLGG from multiple countries, objective responses were observed in 80%, after discontinuation 76.5% experienced rapid progression (median 2,3 months), but upon rechallenge with vemurafenib (± a MEK-inhibitor), 90% achieved an objective response [11].

Other authors have already reported on clinical responses to vemurafenib, but no one assessed an imaging improvement earlier than two months after the start of the therapy [10, 25].

In conclusion, we report the abrupt radiological response (correlated to neurological improvement), after only 6 days and four weeks of treatment with vemurafenib; the MRI follow-up showed further shrinkage and then stabilization in mass volume. To our knowledge, this is the first report assessing with consecutive MR images such a quick shrinkage in PLGGs treated with vemurafenib, highlighting the importance of an early investigation of BRAF status in all cases of LGG in children. We also wish to underline the pivotal role of neurosurgical biopsy for molecular diagnosis, in order to let a prompt *BRAF* mutations identification and guide subsequent targeted therapies. Being our first objective the rapid relief of symptoms due to tumor compression, such a remarkable result adds an important experience on the sparing of surgery-related procedures. The main limitations of this report are the ones expectedly pertaining to a case description involving a single or few patients, being PLGG a quite rare condition and vemurafenib having been adopted as a possible therapeutic strategy in pediatric tumors for a relatively short period. However, our cases demonstrate how a prompt radiological response to vemurafenib, and the related clinical improvement, can influence both therapeutic and surgical decisions, hopefully reducing the occurrence of second neurosurgery with associated risks of neurological sequelae.

## Data Availability

Data sharing not applicable to this article as no datasets were generated or analysed during the current study.

## Abbreviations

PLGG: Pediatric Low-Grade Gliomas
MRI: Magnetic Resonance Imaging
PFS: Progression-Free Survival
